# Uptake of planning as a self‐regulation strategy: Adolescents’ reasons for (not) planning physical activity in an intervention trial

**DOI:** 10.1111/bjhp.12595

**Published:** 2022-04-22

**Authors:** Elina Renko, Katri Kostamo, Nelli Hankonen

**Affiliations:** ^1^ Faculty of Social Sciences University of Helsinki Helsinki Finland; ^2^ Faculty of Social Sciences Tampere University Tampere Finland

**Keywords:** adolescents, content analysis, Finland, physical activity, planning, self‐regulation

## Abstract

**Objectives:**

Planning is an effective self‐regulation strategy. However, little is known why some people take up planning and some do not. Such understanding would help interventions to promote planning. We investigated how adolescents explain their (non) use of planning for physical activity after an intervention.

**Methods:**

Qualitative content analysis was employed to investigate follow‐up interviews (a purposeful sampling; *n* = 19 low‐to‐moderately active, vocational school students) of Let's Move It trial participants twice post‐intervention: 6–8 weeks and 14 months post‐baseline. In the intervention, planning was one of the key techniques used to promote PA.

**Results:**

We identified seven categories linked to reasons for (not) using planning. Most were related to feelings anticipated to result from planning. Action‐ and identity‐related concerns were also raised. The reasons for planning were that the plan (1) helps to clarify what to do and to get things done, (2) strengthens the feeling of autonomy, (3) promotes a sense of progress, ability and control over one's PA. The reasons for not planning were that (having) a plan may (1) feel forced and like an unpleasant duty, (2) take away life's spontaneity and freedom, (3) result in anticipated annoyance and bad mood if one fails to enact the plan, or (4) be an effective strategy for others but not for the interviewee.

**Conclusions:**

Planning may not only link to behavioural control but also the sense of autonomy, and thus subsequent motivation. We suggest various strategies to promote planning, including challenging non‐planner identity and harnessing social dimension of planning.

**Statement of contribution**

***What is already known on this subject?***

- Planning can be an effective strategy for behaviour change but not all engage in it
- Understanding intervention participants’ views can improve planning uptake

***What does this study add?***

- Adolescents raised action‐ and identity‐related concerns in their accounts of (not) planning
- (Not) planning was explained mostly by anticipated negative or positive feelings
- Planning ahead may strengthen the sense of being able, being in control and making progress

## BACKGROUND

Physical activity (PA) has various health benefits, such as reducing overweight and obesity, improving depressive symptoms and controlling blood pressure, and thus it reduces the risk for non‐communicable diseases (Chaput et al., 2020; Guthold et al., 2018; Janssen & LeBlanc, 2010). Current recommendation for adolescents includes at least 60 min per day of moderate‐to‐vigorous PA and vigorous‐intensity aerobic activities, as well as muscle and bone strengthening activities that should be incorporated at least 3 days per week (Chaput et al., 2020). Despite the recommendations (Chaput et al., 2020), adolescents’ PA levels have remained low (Elgar et al., 2015; van Sluijs et al., 2021) and efficient interventions are needed to improve global PA levels (Hallal et al., 2012). While changes in built environment and subsidies for disadvantaged groups are necessary, an important part of PA promotion includes individual level interventions (Sallis, 2018). In most current societies, physically active chores are not a natural part of daily life, thus, getting sufficient PA for health necessitates self‐regulation. Planning as a self‐regulation strategy is among the most cited and used techniques in health behaviour interventions (Gollwitzer, 2014; Hagger & Luszczynska, 2014; Hagger et al., 2016; Rhodes et al., 2020).

Planning skills are essential to transform abstract goals into action (Schwarzer & Luszczynska, 2008). A vast body of research demonstrates the discrepancy between individuals’ reports of good intentions to engage in health behaviour change and failure to act on those intentions (Rhodes & de Bruijn, 2013; Sheeran & Webb, 2016). This intention–behaviour gap may be due to several reasons and hindrances. Individuals may lack opportunities to get started (problems with action initiation), they may face unforeseen barriers, attractive opportunities, competing goals and temptations (problems with overcoming obstacles), lack the needed effort over time (problems with persistence) (Hagger et al., 2016; Heckhausen, 1991; Rhodes et al., 2020) or form intentions that are not sufficiently specified (Schwarzer, 2008). Strategic planning may facilitate bridging the intention–behaviour gap (Schwarzer & Luszczynska, 2008).

Definitions of planning constructs vary considerably in the literature (Hagger & Luszczynska, 2014; Rhodes et al., 2020) and the debate surrounding the conceptualizations reflects different theoretical and epistemological standpoints (Hagger et al., 2016; Schwarzer & Luszczynska, 2008). According to Rhodes et al. (2020), different types of planning are as follows: *action plans* (how, when and where plans), *preparatory plans* (how plans leading to the action), *implementation intentions* (if‐then plans) and *coping plans* (plans to overcome barriers). Implementation intentions and action plans are two of the most recognized and frequently applied planning techniques adopted to change health behaviour (Adriaanse et al., 2011; Hagger et al., 2016; Rhodes et al., 2020; Webb et al., 2010).

Overall, planning interventions have shown considerable promise, and there is evidence of effectiveness in different behaviours, such as PA (e.g., Carraro & Gaudreau, 2013) or healthy eating (Vilà et al., 2017) and populations (Toli et al., 2016). However, meta‐analyses have identified some heterogeneity in the effects of planning interventions on behavioural outcomes (Bélanger‐Gravel et al., 2013; Carraro & Gaudreau, 2013). Bélanger‐Gravel et al. (2013) conclude that the use of implementation intentions to promote PA was more likely to be effective among students and clinical samples compared with general population of adults. Furthermore, Carraro and Gaudreau (2013) argue that planning is especially beneficial for people with chronic difficulties in regulating their behaviour (see also Gollwitzer & Sheeran, 2006).

We focus on low‐active adolescents’ reasons for planning or not‐planning PA. Adolescence is a developmental period characterized by changes in social and affective processing, cognition and social behaviour. For example, while still influenced by family values adolescents are also oriented to greater autonomy and independence (Dahl et al., 2018) and more drawn to peer groups (Crone & Dahl, 2012). Meanwhile, social evaluation and social status gain more importance (Dahl et al., 2018). Preference for immediate rewards increases from pre‐adolescence, peaks in mid‐adolescence and then declines, whereas brain systems that are active in self‐regulation continue to develop into late adolescence or even young adulthood (Steinberg, 2014), enabling more long‐term, internalized and voluntary goal setting and motivation (Gestsdottir & Lerner, 2008). As such changes offer opportunities for learning and positive motivations (Crone & Dahl, 2012), interventions that acknowledge adolescents’ needs for belonging, respect and admiration, may attain lifelong impacts (Dahl et al., 2018).

There are indications from intervention process evaluations that suboptimal proportion of participants use planning as a behaviour change strategy (e.g., Hankonen et al., 2015, 2017; Hagger et al., 2016; Knittle et al., 2016). Therefore, evidence base is biased in the sense that not all planning interventions have had high ‘enactment fidelity’: This means that even though intervention providers would have successfully prompted participants to create plans (high delivery fidelity), participants have failed to understand instructions (receipt) or enact planning, that is, actually create action plans for themselves (enactment fidelity) (Bellg et al., 2004). High enactment fidelity is a key requirement to a) produce expected outcomes and b) successfully be able to evaluate the effectiveness of the intervention (Hankonen, 2021). Only recently researchers have started to pay attention to this aspect, in addition to facilitator *delivery* of BCTs (behaviour change techniques). Indeed, planning is one of the behaviour change strategies for which participant enactment is crucial for its effectiveness (Hankonen, 2021; Knittle et al., 2020).

There is surprisingly little research into the reasons why some people do take up planning, and why some people do not. Qualitative investigation of these reasons may help to understand how to better support planning and to develop interventions that use planning further. Additionally, such understanding could be useful for researchers wishing to establish effectiveness of planning interventions: For trials to be useful in producing such evidence, intervention arm has to have high fidelity, that is, uptake of planning by participants.

To develop more effective planning interventions for practice and useful trials for research, we need to understand how to best promote the uptake and use of planning as a behaviour change strategy. We propose that such understanding *requires* (1) exploring intervention participants reasons for planning or non‐planning and (2) approaching planning as a behavioural target in itself. This study seeks to capture that by exploring the reasons students give for (non) use of planning to increase their PA.

## METHODS

### Study design

As interviews tend to generate rich data about individual perspectives and allow identifying views that could not have been anticipated (Braun & Clarke, 2006), we chose individual face‐to‐face interviews to illuminate adolescents’ perspectives on (not) planning PA.

The semi‐structured interviews were conducted during a cluster‐randomized trial evaluating effectiveness and processes of the school‐based intervention to promote PA and decrease sedentary behaviour, Let's Move It (LMI) (Hankonen et al., 2016). The activities aiming at increasing moderate and vigorous physical activity (MVPA) were especially targeted for those with low or moderate levels of MVPA at baseline. One main component of the intervention was a six‐session classroom training for students, focusing on fostering motivation and providing self‐regulatory skills for increasing students’ PA in leisure time, with self‐led behavioural experiments of novel forms of PA embedded between sessions. The student intervention component of the LMI drew from self‐determination theory (SDT; Ryan & Deci, 2000), self‐regulation and planning theories (Carver & Scheier, 1982; Hagger et al., 2016) and reasoned action approach (Fishbein & Ajzen, 2011). It provided intervention participants with behaviour change skills to self‐motivate and self‐regulate, for more leisure time PA (Hankonen et al., 2016).

A systematic review (Hynynen et al., 2016) identified planning to be characteristic of effective school‐based interventions to promote PA among older adolescents. During the LMI intervention, planning was promoted and a variety of self‐regulatory strategies were introduced to participants. They familiarized themselves with SMART goal setting (i.e. a goal should be specific, measurable, attainable, relevant and time based) and were guided to plan how, when, where, with whom and why they would be physically active. Furthermore, several self‐motivation techniques were taught (e.g., finding one's own reasons to be physically active, seeking social support) and PA self‐monitoring tools provided (e.g., a self‐chosen mobile app or a paper‐and‐pencil diary to record PA sessions). The full intervention content is described in detail elsewhere (Hankonen et al., 2020).

As part of the mixed‐methods evaluation of the trial (see Hynynen et al., 2016; Heino et al., 2019), subsample of students in both trial arms were interviewed. The interviews took place after the intensive intervention at 6–8 weeks post‐baseline (see also Kostamo et al., 2019; Palsola et al., 2020), and 14 months after baseline.

### Participants

The main LMI trial included six schools (Hynynen et al., 2016), at baseline 528 control arm and 638 intervention arm students participated (Heino et al., 2019). The control condition received teaching as usual. A purposeful sampling was used to invite a subsample of participants (*n* = 34) for interviews. The invited participants were previously low‐to‐moderately active (based on students’ self‐reports at baseline), 15‐ to 19‐year‐old vocational school students who were studying a basic vocational degree, had minimum 4/5 self‐assessed skills in Finnish language, no background in competitive sports, at least four LMI sessions attended and signed consent for the study. All students who had participated in the first post‐intervention interviews (6–8 weeks post‐baseline, T3) were invited for follow‐up interviews (14 months after baseline, T4), 19 students agreed to participate both interviews. This analysis includes only those interviewees that attended both interviews. Table 1 demonstrates the distribution of participants in regard to gender, school and educational track.

**TABLE 1:** Description of interview participants at both follow‐ups

	Participants in intervention schools	Participants in control schools
Practical nurse students	10 (9 women, 1 man)	3 (2 women, 1 man)
Hotel, restaurant and catering students	4 (3 women, 1 man)	2 (2 women, 0 men)
In total	14 (12 women, 2 men)	5 (4 women, 1 man)

Participants were recruited through research assistants face‐to‐face or via phone. All participants received study information sheets and gave a written consent. As a compensation for their time, participants were offered a movie voucher. The study procedures were reviewed by the ethical committee of the Hospital District of Helsinki and Uusimaa (367/13/03/03/2014).

### Procedure

The interview duration ranged from 24 to 80 min. The interviews were carried out by three trained research assistants at school on students’ free time. All interviews were audio‐taped and transcribed verbatim. An interview topic guide (see: OSF link & OSF link) ensured consistency across interviews and consisted of four main topics. This analysis focuses on the topics 2 (Strategies to increase, maintain or manage PA) and 4 (LMI intervention program), which best invited commentary around planning. Topics 1 (Changes in PA behaviour) and 3 (PA‐related thoughts) have been analysed and reported elsewhere (see Kostamo et al., 2019). Concerning planning, the participants were asked to what extent they plan their PA, how they motivate themselves, whether they pay attention to their amount of PA, what kind of PA goals they have and how they monitor their PA goal achievement. Questions focusing on self‐regulatory technique enactment were selected specifically, because observations in this and previous interventions indicate that planning is not easily taken up.

Planning‐related questions and comments were present in both datasets (post‐intervention interviews T3 & follow‐up interviews T4). Based on experiences from the T3 interviews, for the T4 interviews the interviewers were advised to ask more "why" and "how" questions, especially in regard to planning and other self‐regulatory techniques. Thus, final follow‐up interviews focused particularly on this matter, asking participants more about the reasons behind (not) planning. Consequently, the interview talk concerning planning was richer in the T4 than T3 interviews and we decided to start our analysis focusing first on the T4 interviews, and only then on the T3 interviews.

### Analysis

We employ qualitative content analysis. This method can be described as the systematic analysis of texts focusing not only on manifest content but also latent content (that may not be overtly and literally evident in a communication). As qualitative research generally, qualitative content analysis does not seek to be universally applicable but meaningful in context (Drisko & Maschi, 2015; Mayring, 2000).

First, the authors (NH and KK) and an external researcher discussed research questions and data analysis strategies. A collaborative approach was agreed upon, in that two researchers (ER and KK) would both independently familiarize themselves with the data and then compare notes in a collective discussion. ER and KK used inductive content analysis to familiarize themselves with the data by reading it through and noting down initial ideas about PA change processes and the uptake of behaviour self‐management strategies. Next, KK condensed patterns of shared meaning concerning PA change and use of self‐management strategies and gave them to ER for checking. This was followed by a collaborative phase, KK and ER organized the patterns of shared meaning into potential categories. By involving more than one researcher in the reflexive analytic process, we aimed to develop richer reading of the data (see Braun & Clarke, 2019). Related to self‐management strategies that adolescents report using, categorization resulted in three subgroups: (1) active cognitive planning, (2) using daily habits and rhythm and (3) strengthening social support. These were discussed amongst all authors. After this, KK and ER refined the research questions in the light of preliminary findings and proceeded to analyse the data independently: ER focused on the interview talk concerning planning PA (this manuscript).

The final research question this study explores was shaped and crafted during the following inductive content analysis that ER performed. The analysis revealed that comments about planning were often contradictory: The same interviewee could describe doing quite precise plans (implementation intentions, action planning, etc.) for PA and on the other hand, that he/she does not actually plan PA at all. ER continued analysis to explore further, how adolescents explained their (non) use of planning for PA. She analysed all the reasons given for (not) planning PA and classified them into reason categories. All the given reasons fitted into these categories. The first draft of the reason categories was reviewed with co‐authors. After this, the reason categories were refined, including the final names for each category. Finally, each participants’ reasons were mapped separately in order to see whether reasons given for (not) planning were similar in intervention and control groups.

## RESULTS

In the analysis, we formed three categories of explanations the interviewees gave for planning PA. These reason categories highlight the benefits that adolescents saw in implementing planning strategies to facilitate behaviour change. Reasons for planning were similar in intervention and control groups, and as no major differences between the groups emerged, the findings are presented in the entire trial cohort. Also, categories formed from the interview data collected at both follow‐up points did not differ.

### Reasons given for planning PA

#### Planning ahead: to clarify to oneself what to do and to get things done

Accounts in this category illustrated planning as useful in the sense that a plan will be carried out once it is made. With a plan, one knows what to do, without additional reflection or cognitive burden and one must stick to the plan.

SH7 (T3): And then it'll be like, you do the thing.

SH6 (T4): I plan the physical activities that I’m gonna do, so it goes much easier, so that I don't have to start thinking on the spot what I should do.

SH8 (T4): Like when you have made an agreement with yourself or with friends or whoever you're going with, then you just have to go.

However, some interviewees emphasized that the plan supports action only when it had been prepared with one's personal life situation and other daily schedules in mind.

SH3 (T3): I think how it'd fit my schedule. Like when would I do it, should it be in morning or afternoon or evening, that's something I gotta think beforehand.

SH5 (T4): I check that I take my stuff [the right gear for PA] to school and I’ll just go straight to the gym, and then straight to work, just to make sure it's all done.

Moreover, the interviewees emphasized social dimensions of planning. A plan drawn up together must be followed for the sake of others, and they also remind about it.

SH15 (T4): If I plan a thing, I plan it with some friends, because then I have somebody to remind me that hey, today is the thing, and then I have more willpower for it than when planning alone, like if I plan alone then it's easier to fail to go just because I don't really feel like it.

Also, some mentioned that to get things done with friends, simply picking a time would be sufficient planning. The details regarding being physically active would then be figured out together.

SH16 (T3): I mean, it's like when you come to school in the morning and when you are like, actually awake, someone comes ask you like “hey wanna go and play something today” and then I’ll be like “all right message me on Whatsapp” and we'll pick a time and everyone will be there. (‡) It always works well like that, no need to plan anything, we'll just go hang out on the street and something will always pop up. That's how great these guys are.

#### Planning ahead: to strengthen the feeling of autonomy

The interviewees highlighted that a self‐made plan strengthens the feeling of autonomy: Deciding personally how to move and having the power to also flexibly modify the plan if necessary. A self‐constructed plan can also be changed freely.

SH13 (T4): I have like this diary, where I write up what I will do on weekly basis. So I can divert a little [from the plan], or change what I do, as like for example I scaled down a bit the routines that I used to do, so I still have energy to go to work.

SH6 (T3): I mean, I’d follow the plan, but at the same time, I’d change some things as well. If I have long days at school, I’d like to take, you know, a small nap, then I’d wake up and do some jogging, for instance.

#### Planning ahead: to achieve personal pa goals and to strengthen the feeling of progress

Within this category, the interviewees stressed that planning strengthens the feeling of control and gives an actual sense of progress and ability. Monitoring personal improvement becomes easier when one knows what one was supposed to do and strive for.

SH5 (T4): I have different kind of days, like a day can be, for example, a leg day, a stomach day, an upper body day, or cardio, so that you are training your whole body depending on what kind of day it is.

SH16 (T3): I’d definitely wanna see how I’m getting better.

### Reasons given for not planning PA

For not planning PA, the analysis created four categories. These categories were similar in intervention and control groups and in the interview data collected at two time points.

#### Not planning: to avoid the sense of obligation

Many interviewees noted that planning can also generate feelings of being under pressure. They described that a self‐created plan may feel like a commitment causing stress and anxiety. Doing PA should feel like a fun pastime; however, if the planned activities lead to the sense of obligation you are forced to fulfil them and it takes all the fun out of them.

SH2 (T3): Stressful. Uncomfortable. It's like, I gotta commit. Like I’d way rather do it when I’m in the right mood, that I wanna do it, and that it feels fun.

SH6 (T4): Because it's not fun if you always plan the stuff to the end and make it into a schedule as if it was in school or at work. It should anyway be fun and something you want to do.

#### Not planning: in favour of a sense of spontaneity

The interviewees often expressed that planning takes away the spontaneity and freedom in decision‐making – as if the plan was forcing and ordering from above. They pointed out that planning may clash with their desire to ‘live in the moment’.

SH1 (T3): I’m not really that interested in planning stuff, like if I wanna do it I’ll do it.

SH16 (T4): I don't like it myself. As an example, if I go skating, I do what I like to do, it's not like that now I’ll do sprints between lines for ten minutes. I just do what I feel like doing.

Accounts in this category emphasized that PA happens on its own, without thinking or planning. PA was illustrated as an area of freedom, in which planning has no role – plans would only make the situation more cumbersome.

SH2 (T4): I don't really think about it though. Or you know, think about it that hard. I just do my thing.

SH1 (T3): I guess it's nice that there's more freedom in one area of your life [doing PA].

‘Not planning in favor of a sense of spontaneity’ and ‘not planning to avoid the sense of obligation’ were often intertwined and some reasons for not planning fitted to both categories.

SH16 (T3): Dunno, maybe it just starts stressing you out like ‘damn, I had promised to go there tomorrow’. I just like living in the moment. I guess. It's just my thing. So that [planning] doesn't sound good at all.

SH17 (T4): Well, if I make it [the plan], then I get the feeling that I really need to do it, and that sometimes even brings me anxiety. That I like to go according to my moods, like that the mood I’m in determines [the flow of the workout] as well, like, I have no need to plan it ahead in such detail.

#### Not planning: in order to avoid a feeling of failure, annoyance and bad mood

Some interviewees reported that if the execution of the plan fails, annoyance and a bad mood follow; ‘I could not do it’.

SH18 (T4): I don't like to make plans myself, like if you make one and fail to follow it, it'll just make you feel bad.

SH10 (T3): I think it's a bit funny, or like, kinda weird cuz you never know what's going on. If we have planned something and then something else comes up, it's just really annoying.

SH13 (T4): If I plan that at four o'clock I will do this, this and this, and if I am not there doing those things at that time, that kind of makes me annoyed, because you'd made a plan and if you're not for example at home then [when you were supposed to], then you feel kind of like, that I failed.

In addition to own frustration, cancelling a plan may also cause feelings of confusion and uncertainty in other people as well. However, a single light‐hearted planned attempt to do PA might not cause such feelings even if the execution of the plan fails.

SH16 (T3): If you start planning all that stuff, like sure I’ll go there tomorrow with you, and then it gets cancelled, that would cause lots of hassle. I don't like it.

SH2 (T3): If we're like trying some new thing and make a plan for that in advance, then it's not really that bad, but if it's always the same thing, like I have to be somewhere at certain time, then it's like, can I make it.

Some interviewees considered that making ‘small plans’ suited them because they are ‘lighter’ and attainable. However, large‐scale planning was considered unrealistic and unsuitable.

SH1 (T4): I have noticed ages ago, like years ago, that for me the precise plans do not really work, like they fail like after a minute or so already. So, I like to make like …. like a small plan.

SH19 (T4): Well, it depends, if it is something lighter then I can plan it, like that on Saturday I’ll go for a long walk with my dog or something or that I will ask my mum to go with me, those kinds of things I might plan ahead.

#### Not planning: because it is not ‘my cup of tea’

Often, the interviewees brought up that planning may work for others but not for me because I'm not a “planner type”. Planning was illustrated as beneficial in general but not useful or suitable for themselves. Some participants described optimal enactment of planning but still paradoxically stated that “detailed planning does not suit me”.

SH10 (T4): I am really not just that kind of type, that I like to do everything a bit ex tempore, it's easier to come up with something when you haven't though everything through completely, like what to do and when. It's easier to think like “hey, lets do this” and “lets do that” and then it is also easier to make it happen in my opinion.

To justify this view, the interviewees pointed out that they themselves lack the characteristics that are needed to get benefits from planning. In particular, planning would work for those who use a calendar, remembered things well and like to sit down to think.

SH2 (T3): I mean, I’m sure it'll work for someone who uses a calendar or something, you can just check there and all that, but the thing is that I don't use a calendar or anything like that cuz I still won't remember and then it'll be like damn, I forgot to write down all this stuff again.

SH18 (T3): Like, it takes time. And I’m not that kinda guy to sit down and start planning all these things, I’ll just do it. Or I guess I just like doing things more than thinking.

Some interviewees mentioned considering themselves free spirits who do not need planning.

SH5 (T3): It's pretty nice. But not really my thing honestly. I like to be free.

Furthermore, they noted that planning would be more suitable for athletes who train seriously.

SH7 (T4): It would suit better for athletes. Like regular gym users, football players or basketball players, but if you're just one who likes to walk or to go to gym every now and then, or stuff like that, it doesn't take special planning, you just go if you feel like going.

SH16 (T4): Since I do not train in a proper team or anything, it is not so serious or anything, so that is why I do not like, I just don't like to plan anything.

## DISCUSSION

This study set out to understand adolescents’ insights and given reasons for taking up planning for PA or failing to do so. To summarize, the main reasons the interviewees gave for planning were that a plan helps (1) to clarify what to do and stick with it (especially when planning involves social dimensions), and (2) in strengthening the feeling of autonomy as well as (3) to achieve personal PA goals and to strengthen the feeling of progress over one's PA. The first of the reason categories focuses on the beneficial *acts* that planning results in (helps to get started with goal striving and to stay on track, thus solving the main types of volitional problems cf. Heckhausen, 1991), whereas the second and third reason categories highlight the positive *feelings* that planning gives rise to. The reasons the interviewees expressed for not planning were that a plan may: (1) feel forced and like a duty, (2) hinder the feeling of spontaneity and freedom, (3) result in annoyance and bad mood in case it fails and (4) be suitable for others (especially athletes) but not for the interviewee. The three first categories highlight that planning might give rise to negative *feelings*, while the fourth category emphasizes *identity*‐*related dimensions* of planning. Table 2 summarizes the main findings and presents implications for interventions based on the findings. We elaborate on these ideas below.

**TABLE 2:** Summary of the results and suggested implication for planning interventions

Summary of results	Strategies to support planning based on the findings
**Participants’ reasons for planning**	**How to harness these positives to promote planning**
1. Clarify what to do and to get things done ‐ *Automaticity:* Plans as guides, giving clarity (no cognitive burden of choices/decisions), works if taking life situations into account and making necessary preparations *(requires creativeness and effort!)* ‐ *Social dimensions (collaborative planning)*: You cannot let a friend down by failing to enact the plan, friends remind you ‐ *Often even partial planning works (e.g., picking a time)*	‐ Provide these experiences as examples of how others have experienced benefits of planning (modelling) ‐ Make sure all participants understand the mechanisms by which planning aids (e.g., you do not have to consciously think about what to do, just rely on what your previous self decided) ‐ Make sure participants have sufficient time to make preparations and fit the plans to match their life requirements ‐ If possible, support dyadic or collaborative planning
2. For a sense of ability, control and progress ‐ Combines planning with *BCT self‐monitoring & reviewing progress*	‐ Provide these experiences as examples of how others have experienced benefits of planning (modelling) ‐ Highlight the usefulness of planning in progress monitoring (when combined with self‐monitoring)
3. For a sense of autonomy ‐ I make plans myself ‐ I can flexibly modify plans, I am not prisoner of the plan → combines planning with *BCT reviewing goals*	‐ Provide these experiences as examples of how others have experienced benefits of planning (modelling) ‐ Highlight that YOU are the one making plans ‐ Highlight flexibility and permission to modify plans if needed (modified plan is not a failure!)
**Participants’ reasons for not planning**	**How to tackle these challenges/barriers to planning**
1. Plans feel forced and unpleasant duties causing stress and anxiety *Related beliefs or misunderstandings:* “Plans are not flexible, they make PA feel like duty such as school or work, and PA should not feel like that, but be fun”	‐ Manage/correct misunderstandings around rigidity of plans ‐ Support autonomy during the planning process ‐ Offer possibilities for reviewing and modifying plans ‐ Find a middle ground between too vague and too specific plan
2. Plans take away life's spontaneity and freedom	‐ Offer possibilities that may be particularly suitable for those who value spontaneity: Making use of opportunities when they arise
3. Plans risk not being realized, causing annoyance, bad mood and feeling of failure (lowering self‐efficacy) ‐ Cancelling a plan may cause feelings of confusion and uncertainty in others as well ‐ Lighter, imprecise, attainable plans are seen as acceptable, but heavy, large‐scale plans not *Related beliefs or misunderstandings*: “If you make a plan, then you should inflexibly follow it, no excuses, otherwise you are a person who failed”	‐ Manage/correct misunderstandings around rigidity of plans and planning: Highlight the need of allowing some flexibility in the planned activities ‐ Form coping plans and create other tools that may support planning self‐efficacy. ‐ Support dyadic/collaborative planning with, for example, friends, as people report feeling more likely to not fail to enact plans when they were made with friends ‐ Relapse prevention: Normalize “failure”, teach participants to attribute causes of non‐realized plans to external, unstable causes rather to one's fixed traits
4. Plans are not “my cup of tea” (identity, self‐concept) ‐ Planner type: Personality (only people with certain characteristics benefit from planning) ‐ I am “Free spirit” who does not need planning ‐ Planning is more suitable for athletes with training schedule	‐ Challenge non‐planner identity ‐ Emphasize the variety of ways to plan ‐ Manage/correct misunderstandings regarding mismatch between planning and one's self‐concept

### Reflections on the findings

Interestingly, the reasons given for both planning and not planning emphasized the feelings that planning may result in. These feelings reflect the basic psychological needs of autonomy and competence that SDT highlights (Ryan & Deci, 2000). SDT suggest that nurturing the needs of autonomy, competence and relatedness fosters autonomous and internalized motivation. An autonomously motivated individual performs an activity for its own sake, or because it is deemed personally meaningful, resulting in high‐quality behaviour. The results indicate that planning can either support or thwart the basic psychological needs of competence and autonomy (i.e. either by strengthening the feeling of control, progress, ability and autonomy or by promoting the feeling of failure, of being obliged and unspontaneous).

These results have a high relevance for health promotion interventions. Recognizing the feelings that planning associates with is important since needs satisfaction is shown to foster autonomous motivation, and autonomous motivation is strongly related to engagement in PA (Su & Reeve, 2011). Thus, how to create a local planning intervention context that facilitates the fulfilment of basic needs and promotes planning that would maximize experiences of autonomy and competence for all participants? In line with SDT, some would suggest that plans should be self‐formed, rather than formed by someone else, or predetermined, and that planned actions should fit a person's own resources, lifestyle, goals and priorities. However, as interviewees pointed out, even a self‐developed plan can fail or start to feel as if it would force and command from above, thus thwarting the sense of competence and autonomy.

Planning PA is a complex endeavour (Keller et al., 2017). The reasons vocational school students give for (non) use of planning demonstrate well the complexity of planning activity in the context of PA promotion. As described above, the reason categories highlight the feelings that planning may result in. Furthermore, investigating the reasons for (not) planning sheds light on the interviewees’ views concerning barriers and facilitators to planning as a behavioural target in itself. The reason categories have implications to practise and create better understanding of how to better motivate, support and maintain the use of planning in PA promotion (see Table 2). Looking at planning as a behavioural target in itself is important since it leads us to ask what ‘meta behaviour change techniques’ or intervention‐delivery techniques could be effective in motivating, supporting and maintaining the use of planning (see Hankonen et al., 2015; Greaves, 2015).

Regarding facilitating influences, our results highlight social dimensions of planning. Interviewees mentioned that sharing goals and discussing plans with someone made plans more memorable and relevant. These results are in line with findings indicating that dyadic/collaborative planning is linked with higher plan enactment rates than individual planning (Hagger & Luszczynska, 2014; Keller et al., 2017; Luszczynska et al., 2006). Overall, the findings confirm the importance of *taking the social dimension of planning into account*. Theoretically, highlighting social dimensions and involvement of significant others in the individuals’ planning may address the basic psychological need of relatedness (Ryan & Deci, 2000). Indeed, lately it has been noted that behaviour change research has had an unduly large focus on individual, intrapersonal processes, despite the considerably high relevance of interpersonal processes (Rothman et al., 2020). Many BCTs, beyond planning, may be more efficacious when enacted collaboratively. For instance, there is meta‐analytical evidence that goal setting results in better results in teams/dyads compared to individual goal setting (Epton et al., 2017). However, our findings indicate that cooperative planning and planning with friends can be perceived not only useful but also as stressful – if the execution of the plan fails feelings of confusion and uncertainty follow in other people as well.

Regarding barriers, there appeared to be a discrepancy between embracing the importance of planning in general and perceiving it useful for oneself. While adolescents often saw benefits in planning, concerns were raised regarding whether planning fits together with their non‐planner identity. Some interviewees highlighted that the degree to which people benefit from planning differs per individual (see also Palsola et al., 2020). The mentioned reasons for this were related to identity considerations, such as identifying oneself as ‘a free spirit’ that simply does not plan anything. This is in line with evidence which suggests that implementation intentions are less beneficial for those individuals who report high levels of impulsivity (Churchill & Jessop, 2010).

Acceptability of planning and other BCTs may partly depend on personality and other individual difference factors. Thus, *challenging non*‐*planner identity* could be valuable for successful implementation of planning. Additionally, *emphasizing the variety of ways to plan* may be important.

Past research shows that incorporating routines as contextual cues is positively related to PA plan enactment, whereas high specificity of time‐related cues show a negative relationship. This might be because highly specific when cues could be difficult to enact as the specific planned time point is not necessarily suitable for PA and is easy to miss (Keller et al., 2017). In the present study, the interviewees reported that plan execution failures are often followed by “I could not do it” feeling (noted also briefly in Palsola et al., 2020). Further, avoiding such feeling of failure, annoyance and bad mood was reported as a reason for not planning at all. These results demonstrate the importance of *dealing with the fear of not being able to fulfil the plan*. To do so, it could be essential to highlight the need of *allowing some flexibility in the planned activities* (Fleig et al., 2017), forming coping plans and creating other tools that may *support planning self*‐*efficacy*. Besides the results highlight the need of *preparatory planning*; Plans should be prepared with one's personal schedules and life situation in mind. Reflecting “when” and “where” are preparatory actions required to achieve the target behaviour (see Rhodes et al., 2020).

Our results suggest that the act of planning one's actions can both support and thwart the basic need of autonomy. An interesting parallel is research showing that prior action plans can reduce the experience of agency, possibly because action plans could increase the relative automaticity of action (Damen et al., 2015). In our study, the interviewees pointed out that planning can hinder a sense of spontaneity and freedom and the plan itself can start to feel forced and like a duty. To optimize interventions, it would be thus important to support autonomy during the planning process. *Offering possibilities for reviewing and modifying plans* could be essential to increase experienced autonomy in the planned activities. Overall, finding a middle ground between too vague and too specific plan could aid successful plan enactment (see Fleig et al., 2017).

### Implications for practice

Above, we have presented a set of practical suggestions on ways to promote the uptake and sustained use of planning (see Table 2). For example, it could be essential to challenge non‐planner identity (for instance, by emphasizing that different ways of planning exist and by asking; ‘what type of planner are you?’, and providing alternative styles of planning that fit also ‘the wild, spontaneous’ identities). Furthermore, it could be useful to highlight the power of social support, importance of flexible step‐by‐step planning and include planning components to promote self‐efficacy. It should be noted, however, that the reason categories identified in this study may be particular to the age group and other contextual factors here. In interventions, it is always useful to do formative research among the target group to identify particular, specific concerns that may influence *their* use of planning (or other behaviour change techniques) in the intended intervention, and then modify the intervention accordingly (Hankonen & Hardeman, 2020).

In addition, the results shed light on the contexts in which planning may be perceived more effective. When the interviewees gave reasons for not planning PA, they often talked about PA as a fun, flexible, social activity that required spontaneity. In these comments, planning was often viewed as something for athletes or purposefully exercising people do. On the contrary, in the comments that endorsed planning, PA was talked about as a structured and target‐oriented exercise. These comments demonstrate how goal‐oriented behaviour may lend itself to planning much more than general PA that is often a by‐product of something else the adolescents do – such as hanging out with friends. All in all, the results indicate that individual adolescents causality orientation (i.e. autonomy, control and impersonal orientation that, according to SDT, have generalized effects of the orientations on motivation and behaviour, see Hagger & Hamilton, 2021), as well as reasons and goals for doing PA may be factors that determine whether the basic psychological needs are supported or thwarted in the planning process. Independent exercisers may value the possibility to plan themselves when and how to exercise, while hanging out with friends can be more important for those with a non‐planner identity (c.f. Liimakka et al., 2013).

### Reflections on limitations and strengths

This study has several strengths. It provides novel, in‐depth knowledge about adolescents’ insights on planning PA. Research process was marked by reflexivity and the analyses reshaped the research questions to a focus on exploring reasons planners give for (not) planning PA. Approaching planning as a behavioural target in itself is essential to optimize future PA promotion interventions: The findings indicate various strategies that could be effective in motivating and supporting the use of planning.

Another strength is the longitudinal approach: studying the same participants’ accounts and explanations in two different time points. Having rich text accounts from 19 participants in two different interview occasions strengthens and enriches the data even if no major differences emerged between accounts at the two time points. However, one limitation is relying on participants’ verbal accounts of their planning experiences only: Future studies could benefit from combining actual planning records, and quantitative data on participants’ planning activities over the course of intervention, as well as mediating variables. Furthermore, future research should further examine whether adolescents or other individuals who reject the idea of planning actually endorse the same reasons for doing PA. It is prudent to acknowledge that there is no need to push planning on those who do fine without it, and that other BCTs may be more effective for those with a non‐planner identity.

To provide in‐depth understanding of complex phenomena, research needs to take into account the contexts in which individuals or groups function. While looking back at the results, it is important to remember that the reason categories were formed in the context of interviews between trained interviewees and vocational school students who participated in this intervention and, according to self‐reports, were low‐to‐moderately active. Interviewers stressed explicitly confidentiality to the participants as well as the value of different kinds of views. Still the results of any interview study should be interpreted into their social context. In the future, facilitators and barriers of uptake of planning should be studied in other intervention contexts.

Qualitative research is about interpreting and creating – meaning and meaning‐making that is always context‐bound, positioned and situated. We aimed to provide an in‐depth description of the participants’ contexts where we render their insights meaningful (see p. 8–9 for more details). We hope this helps our readers to consider whether and how the results may be transferred to their contexts (Braun & Clarke, 2019; Korstjens & Moser, 2017).

## CONCLUSIONS

This study has explored participants’ own explanations for the use and non‐use of planning for behaviour change. In sum, planning may affect not only concrete abilities and behavioural control, but also the feeling of competence and autonomy experienced, and thus motivation, in various ways. Understanding participants’ perspective will offer important information to improve both planning interventions in practice, as well as fidelity in trials.

## CONFLICT OF INTEREST

No conflict of interest.

## AUTHOR CONTRIBUTION

**Elina Renko:** Conceptualization; Data curation; Formal analysis; Investigation; Methodology; Project administration; Writing – original draft; Writing – review & editing. **Katri Kostamo:** Conceptualization; Data curation; Investigation; Methodology; Writing – review & editing. **Nelli Hankonen:** Conceptualization; Data curation; Funding acquisition; Investigation; Project administration; Supervision; Writing – review & editing.

## Data Availability

The data that support the findings of this study are available from the Finnish Social Science Data Archive (FSD): FSD3437 Ammattiin opiskelevien liikunnan lisäämisen ja istumisen vähentämisen interventiotutkimus: opiskelijahaastattelut 2015–2017: ammattiin opiskelevat henkilöt.
